# Editorial: New insight into the roles of microorganisms in municipal and environmental engineering technologies/systems

**DOI:** 10.3389/fmicb.2023.1349701

**Published:** 2024-01-05

**Authors:** Xiaochen Chen, Ikuro Kasuga, Xianhua Liu, Hongbo Li, Ming Zeng, Xiaolin Cai

**Affiliations:** ^1^Innovation Center for Soil Remediation and Restoration Technologies, College of Environment and Safety Engineering, Fuzhou University, Fuzhou, Fujian, China; ^2^Department of Urban Engineering, Graduate School of Engineering, The University of Tokyo, Tokyo, Japan; ^3^School of Environmental Science and Engineering, Tianjin University, Tianjin, China; ^4^School of Environment, Nanjing University, Nanjing, China; ^5^College of Marine and Environmental Sciences, Tianjin University of Science and Technology, Tianjin, China; ^6^College of Resources and Environment, University of Chinese Academy of Sciences, Beijing, China

**Keywords:** municipal engineering, environmental engineering, microorganisms, conventional contaminants, emerging contaminants, advanced technologies/systems

Aiming at promoting human wellbeing, the municipal and environmental scientists and engineers have developed many technologies and systems to deal with a variety of contaminants (e.g., nutrients, heavy metals, organic contaminants, and pathogens) existing in our ambient environment, in which microorganisms play important roles. On the one hand, microorganisms act a leading role, contributing to the degradation and transformation of the contaminants via metabolism. On the other hand, as a part of the targeted environments or even targeted pollutants, the passive microbial communities can actively sense and respond to the environmental changes, which reflects the performance of the applied technologies/systems or their influences on the health of ecosystems and living organisms.

As modernization, urbanization and industrialization prevail, the environmental quality of the earth keeps on deteriorating. Moreover, some new contaminants come into people's sight, and seem to shift from “unprecedented” to “ubiquitous” overnight, including the latest research hotspots like microplastics (MPs) and antibiotic resistance genes (ARGs). These emerging contaminants and the conventional contaminants could independently or interactively challenge the existing engineering technologies and systems by increasing the level of uncertainty and the risks of malfunction and failure (Bayabil et al., 2022; Puri et al., 2023). Hence, it is very crucial to keep our knowledge about the roles of related microorganisms up to date, so that guidance on the upgrade, optimization and innovation of the technologies/systems could be timely and efficiently offered. Fortunately, a dozen of high-quality papers answered our call-for-papers, and nine are eventually collected in our Research Topic, which cover the Research Topic mainly from three aspects.

The first aspect is the interactions between microbes and contaminants, focusing on mechanistical elucidation. There are three papers involved. Firstly, microbiological nitrogen removal from wastewater is a key issue in municipal and environmental engineering, which in common sense is very susceptible to high concentrations of co-existing heavy metals (HMs). Yang et al. made a breakthrough in advanced wastewater treatment, as a unique aerobic denitrifier was successfully isolated. In addition to efficient auto-aggregating capacity, it showed the potential in simultaneously achieving the nitrogen removal and the biosorption and reduction of highly toxic Cr(VI). Secondly, soil contamination by petroleum hydrocarbons (PHs) is another chronic problem worldwide. Although the PH-degrading bacteria for remediation purpose are available, Zheng X. et al. noticed that the role of microbial ecological processes, including microbial interactions, had been neglected. One of their key findings was that the deterministic assembly of microbial communities mediated the efficient PHs removal. Accordingly, it is recommended to avoid significant soil disturbance during remediation, as directional regulation of microbial ecological functions plays a key role. Thirdly, aerobic composting is a common practice in centralized treatment and resourcilization of manure, while the combined pollution of HMs and ARGs has drawn broad attention. Liu et al. comprehensively studied the interactive mechanisms and environmental drivers of HMs resistome, antibiotic resistance and microbiome. Key findings related to amoxicillin exposure, such as the elevation of HMs and antibiotic resistome, and the discovery of two biomarkers and an important driver, potentially contribute to the synergistic treatment of these pollutants.

The second aspect is microbial sensing and responses to environmental changes, and four papers are involved. Firstly, biofilm universally exists in municipal/environmental water and wastewater treatment processes, and its excessive formation inevitably deteriorates system performance. With a signal compound *cis*-2-Decenoic acid, Song et al. succeeded in controlling the quorum sensing systems and the consequent functions of biofilm microbial communities, including motility, enzyme production, and extracellular polymeric substance. The results contribute to the evaluation on dispersive intensity of biofilm and the relevant biofouling control practices. Secondly, since severe air pollution is caused by straw combustion, straw return on land is highly recommended in the field of agro-environmental engineering. It is worth noting that the interactions between rice roots (rhizosphere) and straw return is likely to significantly influence the establishment of soil microbial community structure and nitrogen cycle, determining soil fertility. Zhao et al.'s study provides a deep insight into the pronounced impacts of straw amendments, and reference for straw return practice. Thirdly, new contaminant MPs widely exist in agroecosystems, but the influencing factors on its geographic distribution, both the biological and the abiotic, remains poorly understood. Yao et al. carried out a case study of five typical rice cropping regions in China, focusing on differences, links and roles of stoichiometric and microbial influences. The findings enrich our knowledge about the toxicology and health risk related to MPs. Lastly, based on synthetic biology (gene transcription), an invention about detecting lead in environmental water was made by Zhu et al. Their dual-color biosensor with direct reading by naked eyes and colorimetric quantification has many advantages in toxic heavy metal monitoring and commercialization potential, such as wide detection (concentration) range, few interfering factors (co-existing metals), user friendliness, and low cost.

The third aspect including two papers is monitoring and control of microbial stability, as well as the relevant environmental and health risks. Red tide caused by dinoflagellates, a form of eutrophication, is threatening water quality and human health of almost all the densely-populated coastal regions. Zheng L. et al. came up with a novel controlling approach, i.e., using the fermentation products from *Pseudomonas* sp. Ps3 strain, which turned out to be very effective in algae-lysis and inhibition in bench scale. In addition, in post-COVID-19 era, public health and safety is still of great concern. Medical wastewater contains a large number of pathogens, and requires careful and effective treatment. Tang et al. composed a comprehensive review on electrochemical disinfection technology for medical wastewater. By reviewing its development status, proposing three-stage system, and discussing its prospects, this paper provide guidance on the research and employment of this promising technology.

As abovementioned, these papers greatly enrich our knowledge about the roles of microorganisms in municipal and environmental engineering. However, as guest editors, we realize that there are still important topics or fields missing, especially for novel approaches for the enhancement of microbial performance, e.g., coupling with functional materials (Echeverria et al., 2020) and rhizoremediation (Kotoky et al., 2018); and bioenvironmental and energy engineering processes for organic waste recycling and agricultural/aquaculture system (Wang et al., 2017). Therefore, Volume II of this Research Topic is recently released, aiming at further assisting the scientists and engineers rise up to the complicated environmental challenges.

## Author contributions

XCh: Writing – original draft, Writing – review & editing. IK: Writing – review & editing. XL: Writing – review & editing. HL: Writing – review & editing. MZ: Writing – review & editing. XCa: Writing – original draft.
